# Instrument Control (iC) – An Open-Source Software to Automate Test Equipment

**DOI:** 10.6028/jres.117.010

**Published:** 2012-05-02

**Authors:** K. P. Pernstich

**Affiliations:** Physical Measurement Laboratory, National Institute of Standards and Technology, Gaithersburg, MD 20899; Institute for Research in Electronics and Applied Physics, University of Maryland, College Park, MD 20742

**Keywords:** Automation, data acquisition, GPIB, Java, open-source, test equipment

## Abstract

It has become common practice to automate data acquisition from programmable instrumentation, and a range of different software solutions fulfill this task. Many routine measurements require sequential processing of certain tasks, for instance to adjust the temperature of a sample stage, take a measurement, and repeat that cycle for other temperatures. This paper introduces an open-source Java program that processes a series of text-based commands that define the measurement sequence. These commands are in an intuitive format which provides great flexibility and allows quick and easy adaptation to various measurement needs. For each of these commands, the iC-framework calls a corresponding Java method that addresses the specified instrument to perform the desired task. The functionality of iC can be extended with minimal programming effort in Java or Python, and new measurement equipment can be addressed by defining new commands in a text file without any programming.

## 1. Introduction

A spectrum of automation software for scientific test equipment is available, ranging from full-fledged solutions that provide data acquisition and management (e.g. LabView, Agilent VEE, and EPICS)^1^ to data visualization and calculus software with the capability to communicate with instruments (e.g. IgorPro, Origin, Matlab, Mathematica, and SciLab), and some measurement instruments even include a text-based script processor in their operating system (e.g. Instrument BASIC [1] or Test Script Processor [2]). This article introduces Instrument Control (iC)^2^ [3], an open-source Java program that provides a framework to automate data acquisition by processing a list of commands stored in a conventional text file. Defining the test sequence with clear text commands is one of the main features of iC as it enables quick and easy adaptation to different measurement needs encountered in day-to-day laboratory situations, and to store the employed measurement sequence together with the measured data for documentation purposes. A distinguishing feature of iC is that new commands for an instrument can simply be defined in a text file which then serves as the “driver” for that instrument. Instrument Control works with General Purpose Interface Bus (GPIB) controllers [4] from major vendors (National Instruments, Agilent, Prologix), it supports the RS232 serial port [5], and support for other communication protocols such as Ethernet, USB, or other proprietary protocols can easily be added. Instrument Control uses Java Native Access [6] to access the platform-specific drivers supplied by the vendors of the communication controller (*.dll, *.dylib, *.so files), and it has been tested on Windows and Macintosh operating systems. Instrument Control is comfortable to use, easily extendable, and it is ideal for somebody already familiar with Java or a similar programming language, or when budgetary considerations or the availability of the source-code are of concern.

The source code including documentation, tutorial videos, and a precompiled version is available free of charge from [3]. The documentation also includes an up-to-date list of supported instruments. The most recent source code can be downloaded from the subversion server into the development environment (e.g. Netbeans) with a single mouse click, and the necessary configuration steps are described in [7]. At the time of writing, the source code comprises 11000 lines of code, and the size of the precompiled version is 12 MBytes. In the following, a brief introduction is presented of how iC is used, as well as an overview how the iC-framework works internally. Section 3 shows that the functionality of iC can be extended with little programming effort in Java and explains how new commands that can be executed from the script can be defined in a *generic* way; i.e., in a simple text file containing the GPIB string to be sent to the instrument and a description of the input parameters for the user.

## 2. Using Instrument Control

**Fig. 1:**
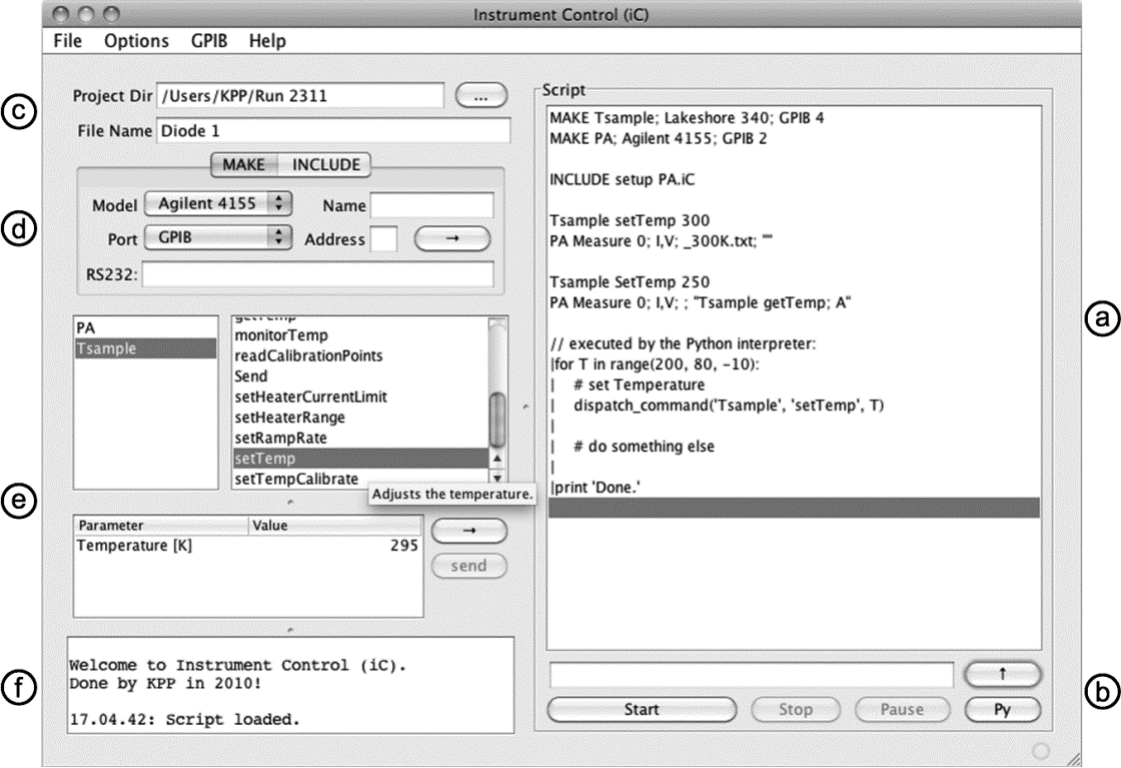
Graphical user interface of Instrument Control (iC).

Central to iC is a *script* (Listing 1) which contains a list of *script-commands*. Scripts are stored as conventional text files, and a graphical user interface (GUI, Fig. 1) implemented in iC offers a convenient way to write such scripts, although any other text editor is also sufficient. Using text files is in general advantageous because text files are universal, cross-platform compatible, unproblematic in terms of long-term readability, and can be read by essentially all programs.

The right side of iC’s user interface displays the script (Fig. 1a), and it contains a line to type in new script-commands and buttons for the user to start, stop, and pause processing of the script as well as to start an interactive Python-Interpreter (Fig. 1b). All measurement data are saved in a project directory and a base file name as specified in GUI (Fig. 1c). Script commands can add an extension to this base file name, e.g., the actual sample temperature when the measurement commenced. The GUI allows the user to add commands to the script that define new instruments (script-command **MAKE**) and to include sub-scripts or Python-scripts (script-command **INCLUDE**, Fig. 1d). Label e in Fig. 1 marks a part of the GUI that lists all instruments defined in the script as well as the commands each instrument supports. This part of the GUI is dynamically generated from annotations in the source code or the text file defining new script-commands in a generic way as shown below. When the user selects a command of a particular instrument, the command’s parameters are shown in a table as illustrated in Fig. 1e for the command **setTemp** of the instrument with the name **Tsample**. This way, the user can add new script-commands with the appropriate parameters. The ‘Send’ button allows the user to send script-commands to the selected instrument while a script is being processed. This is helpful because many instruments cannot be operated from the front-panel while they are accessed remotely. The text-field at the bottom left of the GUI (Fig. 1f) shows status messages for the user.

After the user starts processing the script, all script-commands are parsed to detect errors, for instance typographical errors or parameters that are out of range. This *syntax-check* is performed in the same way the script-commands are executed but without communicating with the instruments, which minimizes the programming effort when extending iC as demonstrated below. After the successful syntax-check, the script-commands are sequentially processed. Each script-command corresponds to a Java method which is invoked by the iC-framework with the proper arguments, or a Python-command that is executed by the integrated Python-Interpreter.

### 2.1 An Example Script

Listing 1 shows an example of an iC-script to measure the current-voltage characteristics of a diode at different temperatures. Lines 1 and 2 define two new instruments: a Lakeshore 340 temperature controller connected via GPIB at address 4, and an Agilent 4155 semiconductor parameter analyzer with GPIB address 2. To refer to these instruments later in the script, the names **Tsample** and **PA** were assigned. Whenever a new instrument is defined, communication with the instrument is automatically established by the iC-framework to minimize the programming effort when the functionality of iC is extended. Line 3 in Listing 1 includes a sub-script, which, as an example, contains script-commands that initialize the parameter analyzer to perform the desired measurements, i.e., assign the source-monitor units, set the range of voltages measured, etc. The next script-command in Line 5 invokes a method **setTemp(float)** in a class which implements all supported script-commands of an instrument (*driver-class*), in this case, the class **Lakeshore340** for the temperature controller. Line 6 calls the method **Agilent 4155. Measure (int, String, String, String)** which starts the measurement and stores the measured parameters I and V in a text file. The name of this file comprises the base file name specified in the GUI (Fig. 1c) and the extension provided in the script-command (Line 6), i.e., ‘Diode 1_300K.txt’. The last argument is optional and allows the user to pass an additional script-command to the **Measure ()** method. This additional script-command (Line 9 in Listing 1) is processed from within **Measure ()** and it’s result is used to attach the current temperature to the file name, e.g. ‘Diode 1_250.15K.txt’.

**Listing 1:**
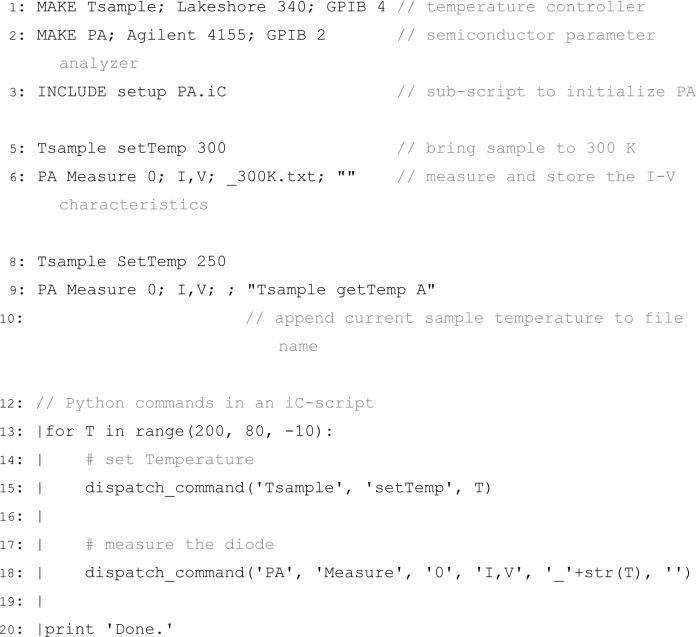
An exemplary script to measure and store current-voltage characteristics of a diode at different temperatures. A line starting with a vertical bar (|) marks Python commands which are executed by the integrated Python-Interpreter.

### 2.2 Python Scripts

To extend the scripting capabilities of iC with variables, loops, conditional statements, and other capabilities of a high-level programming language, support for Python [8] was integrated in iC using the Jython package [9]. Every line in a script starting with a vertical bar (|) is executed by the Python-Interpreter. Lines 13–20 in Listing 1 show Python commands in an iC-script. Line 13 defines a **for**-loop where the variable **T** is decreased from 200 to 80 in steps of 10. The following, indented lines are part of the **for**-loop and executed for each value of **T**. The Python command **dispatch_command (’Tsample’, ’setTemp’, T)** in Line 15 of Listing 1 executes the **setTemp** command of the instrument **Tsample** with the temperature **T**. This command has the same effect as the one in Line 5 of Listing 1 but offers to use a variable instead of a hard-coded temperature. Line 18 measures and saves the diode characteristic and is equivalent to Line 6, again with the use of a variable instead of a hard-coded temperature. After all temperatures are processed, the message ’Done.’ is displayed in a separate window that shows all Python output. This window is also displayed upon pressing the ’Py’ button in the GUI (Fig. 1b), and from this window, Python commands can be executed interactively.

Instrument Control exports a variable _**device** to the Python environment which is an instance of class **Device** and provides access to the iC-framework. A Python script **Startup.py** located in the **<user home>/iC/** directory is executed each time the Python-Interpreter is started and defines the methods **dispatch_command (), is_syntax_check_mode (), is_stop_scripting**, and others. Python commands can also access return values of iC-commands, and more information on the Python integration in Instrument Control is given in the documentation [7].

## 3. Extending Instrument Control (iC)

Instrument Control facilitates three ways of extending its functionality. The programmatic way in which new Java methods are implemented is discussed next, and the generic way of defining new instrument-commands in a text file is discussed subsequently. iC can also be extended using Python as already discussed above.

### 3.1 Extending iC the Programmatic Way

Listing 2 shows a possible implementation of the **Lakeshore340.setTemp** () Java method used to change the temperature set point of a Lakeshore 340 temperature controller and wait until the temperature is within 0.1 K of this set point (a more elaborate version is implemented in iC). The main purpose of this method is to generate the GPIB strings that are sent to the instrument to perform the desired tasks, and to interpret the string which is read back from the instrument that contains the measurement data. Line 5 in Listing 2 creates the GPIB string to set the temperature set point of control loop ‘1’, and Line 8 sends this string to the instrument via GPIB. The method **SendViaGPIB(String**) is defined by the iC-framework and handles all communication with the instruments via GPIB. The method **throws** an **IOException** when a communication error occurs, and this **Exception** is – just as all other **Exceptions** – automatically handled by the iC-framework. Therefore, new code does, in general, not require special **Exception** handling. The documentation [7] elaborates in greater detail how **Exceptions** are used to handle possible errors. In Line 14 of Listing 2, the method **String QueryViaGPIB (String)** is used to query the current temperature of input channel ‘A’. When the difference between set point and current temperature is within 0.1 K or the user has pressed the ‘Stop’ button in the GUI, **setTemp ()** returns and the next script-command is processed.

The documentation [7] contains step-by-step instructions to implement new and extend existing driver-classes, and a reference implementation of a Java method included in the source code is recommended to serve as a template for new code. To minimize programming effort when implementing new script-commands, Java’s Reflection mechanism is used to access class information at run-time. Any **public** method that is added to a driver-class is automatically recognized by the iC-framework and, hence, accessible as script-command without further programming. Java methods are allowed to start new **Threads** to enable parallel processing of certain tasks, such as to display various temperatures on a graph while a script is being processed.

Data received from instruments is in general handled by the Java method querying the instrument, although a return value from a Java method can also be used in the successive script-command including Python commands. Most methods that receive measurement data store the data in a text file for further processing in visualization or calculus software. Java also provides ways to save data as xml files, in a binary format, or in a compressed archive which can be advantageous for large data sets. The open-source software package JFreeChart [10] is integrated in iC to display data in high quality graphs. iC also integrates Apache’s Common Math package [11] for advanced data manipulations such as Spline interpolation of data points, statistical analysis, numerical integration, and much more.

**Listing 2:**
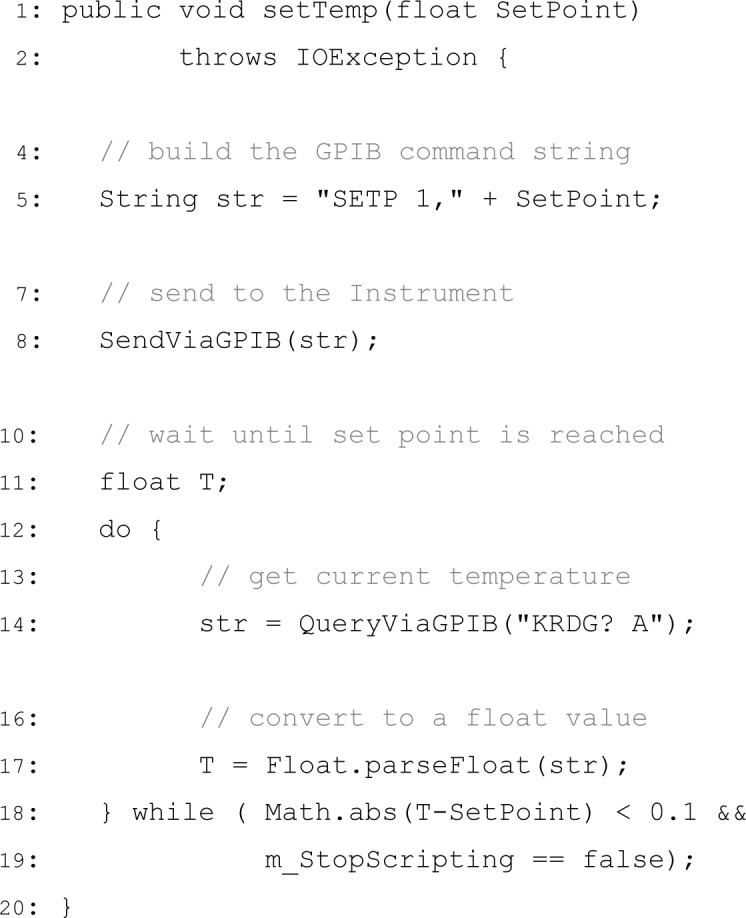
A possible implementation of a Java method to change the temperature set point of a Lakeshore 340 temperature controller and wait until the temperature is reached.

Listing 3 illustrates the mechanism used to dynamically generate the part of the GUI shown in Fig. 1e and also how the syntax-check mechanism is implemented. The Java language allows to define annotations in the source code which can be evaluated at run-time. Instrument Control uses this technique to automatically build a GUI at run-time by defining an annotation **@AutoGUIAnnotation ()** (lines 1–5 in Listing 3) with fields that provide a detailed description of the method’s purpose (tool-tip in Fig. 1e), the names of its input parameters, the default values shown in the table, and the tool-tip texts for each individual parameter.

Instrument Control uses a second annotation **@iC_Annotation ()** (line 6 in Listing 3) which defines if a method performs a syntax-check. If a syntax-check is implemented, the method should throw a **DataFormatException** if a parameter is not allowed (lines 11–12 in Listing 3), and the method must return without any communication when the program is in syntax-check mode (lines 15–16 in Listing 3).

### 3.2 Extending iC the Generic Way

The programmatic way of implementing new script-commands in Java offers great versatility but requires re-compilation of the source code and some programming skills. Defining new script-commands in a generic way using text files requires neither of these and is, therefore, ideally suited for quick testing or implementing simple functions. An example of a generic instrument definition for a SRS Lock-In amplifier is given in Listing 4. The file name (without extension) is taken as the name used in the **MAKE** commands (e.g. **MAKE lia; SRS SR810; GPIB 8**). Every line contains a definition of a new script-command and comprises the following tokens: (1) the name of the script-command; (2) the GPIB string where **%d, %f, %s** (and other format specifiers [7]) are placeholders for integer, double and string values which will be replaced by the values specified in the script; and (3) and (4) specify the parameter names shown in the table of the GUI (Fig. 1e). As illustrated in line 3 of Listing 4, the tokens can also include tool tip texts (enclosed in curly braces) as well as a minimum and maximum numerical value (enclosed in square braces) and a default value for the table (enclosed in round braces). If a method name (token (1) in Listing 4) starts with ‘get’ or ‘save’, the instrument is addressed to talk and the result of this query is made available to the next script-command. If the method name starts with ‘save’ the result is additionally stored in a text file and the last parameter in the generic definition is interpreted as a file extension (token (5) in Listing 4). The text files defining generic instruments need to reside in a particular directory **(<user home>/iC/Generic Instruments/**), the file name must contain ‘.GPIBinstrument’ to be recognized by iC, and it can optionally end with ’.txt’. If the file name matches the name of an existing instrument (e.g. Agilent 4155.GPIBinstrument.txt), the generically defined commands are added to the existing commands of that instrument. All generic instrument definitions are read when iC starts, which makes it very easy to write new “instrument drivers” or extend existing ones without any Java programming.

**Listing 3:**
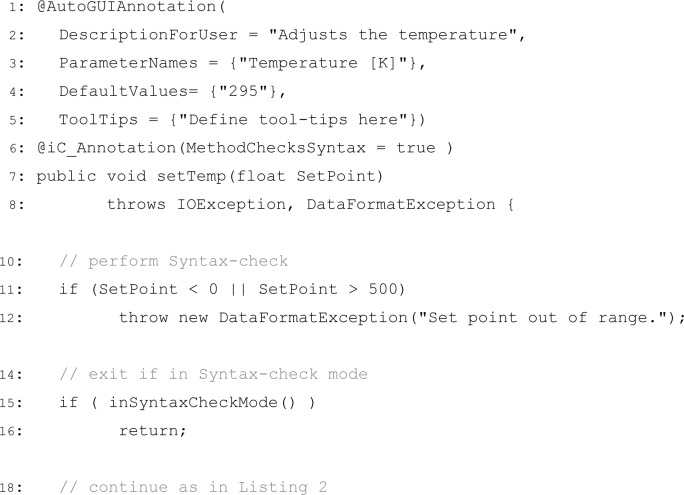
Annotations used by iC to define part of the GUI (Fig. 1e) in the source code, and to declare if a method implements a syntax-check.

**Listing 4:**
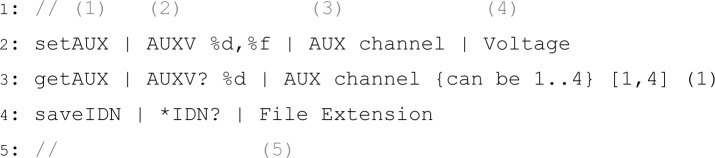
Example for a generic definition of script-commands for a SRS SR810 Lock-In amplifier. These definitions are stored in a text file named ‘SRS SR810.GPIBinstrument.txt’ and automatically recognized by iC at start-up.

## 4. Summary

Instrument Control (iC) is an easy to use open-source software to automate test equipment. iC uses intuitive script-commands stored in a conventional text file to define the measurement sequence which enables quick adaptions to various measurement needs. It is very easy to extend the functionality of iC by either implementing new Java methods in a driver-class, by using the built-in Python-Interpreter, or by defining generic instrument definitions in a text file. Instrument Control works with GPIB controllers of major vendors on various operating systems, and support for other communication protocols can easily be added. Instrument Control is currently used to develop a thermo-magneto-electric measurement platform at NIST, and it is made available as open source to follow a call to publish computer code [12] and in the hope that it will serve the scientific community.

## Footnotes

1 Certain commercial equipment, instruments, software, or materials are identified in this paper to foster understanding. Such identification does not imply recommendation or endorsement by the National Institute of Standards and Technology, nor does it imply that the materials or equipment identified are necessarily the best available for the purpose.

2 The Instrument Control software is an experimental system. Neither NIST, nor the Swiss National Science Foundation nor the author assume any responsibility whatsoever for its use by other parties, and makes no guarantees, expressed or implied, about its quality, reliability, or any other characteristic.
